# *Beyond the Present-Fault Paradigm: Expanding* Mens rea *Definitions in the General Part*

**DOI:** 10.1093/ojls/gqab033

**Published:** 2021-10-30

**Authors:** J J Child, Adrian Hunt

**Affiliations:** 1 Reader in Criminal Law, Birmingham Law School; 2 Senior Lecturer, Birmingham Law School

**Keywords:** *mens rea*, general part, ulterior *mens rea*, complicity, conspiracy

## Abstract

This article explores the use of *mens rea* terms in the criminal general part. We contend the current law fails properly to conceptualise *mens rea* for a large category of offences, namely bespoke/substantive inchoate offences, attempt, conspiracy, assisting and encouraging, and the general offence of complicity. These offences involve two conduct events: one in the present and one in future. However, current *mens rea* terms are defined as if applied to the more conventional category of criminal offence which only involves present conduct—a practice which we term the ‘present-fault paradigm’. We explore the limits of current *mens rea* terms, defined for present-conduct targets (circumstances and results), when applied to future-conduct ulterior targets within inchoate and complicity offences. We contend that current *mens rea* definitions and analysis within the general part are inappropriate for targeting elements related to future conduct/offending, and we suggest more appropriate bases for conceptualising such *mens rea*.

## 1. Introduction

This article explores whether the limited set of general part doctrines recognised within the current criminal law is sufficient to cater for certain offence constructions outside of what we call the ‘present-fault paradigm’. We believe they are not, and that new definitional components within those doctrines are required. This is an argument that ranges across the definitional general part, but will be illustrated in this article through an investigation of *mens rea*.^1^

When considering *mens rea*, at least two points of enquiry should be distinguished. The first relates to the degree or type of culpability established by a *mens rea* term, leading to debates about how such terms should be (re)defined as well as the variety of terms required for special part offences.^2^ This is the more typical focus of academic enquiry.^3^ However, our focus is on a second, distinct point of enquiry: the relationship between *mens rea* terms and the *targets* of those terms, and in particular how the definition of a single *mens rea* term may change or need to be changed depending on its target. For example, ‘recklessness’ as to a *result* element requires the defendant (D) to foresee a risk that her act might cause a particular consequence, whereas recklessness as to a *circumstance* element requires D to foresee the presence of a particular fact without any need to consider causal agency.^4^ Varying definitions in this way, depending on the target of *mens rea*, is a necessary and appropriate recognition of the varying ways in which D interacts with different offence elements.^5^

Academic analysis of this second issue—offence structures and how the targets of *mens rea* may or should affect their definitions—has been limited,^6^ and is rarely expressly addressed by courts. We contend that this is a significant oversight. It creates uncertainty in the analysis of *mens rea* terms in general;^7^ and (our focus here) it has meant the general part of the criminal law fails for the purposes of *mens rea* properly to distinguish between two different offence patterns, which necessitate different approaches to defining and conceptualising relevant *mens rea* terms. The two offence patterns in question are what we call ‘*present-conduct*’ and ‘*future-conduct*’ offences.

*Present-conduct* offence constructions are those most familiar to students of criminal law. These are offences where D completes conduct (consisting of actions or omissions,^8^ in certain circumstances and with certain results) and does so with *mens rea* as to each of these present-conduct elements. *Mens rea* as to each target element must coincide in time with D’s actions, a point we refer to as ‘T1’. For example, liability may turn on whether, at the time D engages in the action, she does so voluntarily and has knowledge of required circumstances, and/or whether, when acting, she intends her action to cause a particular result. Ulterior *mens rea* elements may also come within a present-conduct construction, so long as they again focus on targets from D’s present T1 actions only. An example is criminal damage ‘being reckless as to endangering life’.^9^

*Future-conduct* offence constructions, in contrast, include conduct at T1, but also include a second, later, conduct event (actual or contemplated) to be completed by D or another at a future point in time (we refer to this point as ‘T2’). Such offences still require D’s *mens rea* to coincide in time with her T1 actions, but the targets of that *mens rea* are now spread across distinct future events (ie including the circumstances and results of T2 acts) beyond the traditional elements of present-conduct offences. This multi-event structure, incorporating present and future conduct, is employed for offences across the criminal law. A good example is burglary under section 9(1)(a) of the Theft Act 1968, which criminalises entering a building as a trespasser (ie conduct at T1) with intent to steal, or cause grievous bodily harm or unlawful damage (ie contemplated conduct at T2). Alongside a growing list of similar bespoke/substantive inchoate offences, the same structure is also employed within the general inchoate offences of attempt,^10^ conspiracy^11^ and assisting and encouraging,^12^ and the general offence of complicity, where the T2 conduct must be completed in fact.

We contend in this article that current *mens rea* terms (both in definition and application) are ill-equipped to target T2 elements. Differences between T1 and T2 target elements have been neglected, and present-fault definitions (designed to target T1 elements) are expected to function here without amendment. We call this problem the ‘present-fault paradigm’: where the current law stubbornly attempts to force square present-conduct pegs into round future-conduct holes, creating conceptual and/or normative problems. We contend that new definitions and forms of analysis are required to make sense of *mens rea* for T2 targets.^13^

Section 2 outlines the problem of using *mens rea* terms defined within the present-fault paradigm for future-conduct offences. Our aim here is not to create complexity, but to recognise existing complexities that are hidden or ignored within the current law, and their connection to some of the most (apparently) intractable criminal law problems. Sections 3 and 4 provide separate consideration of two categories of future-conduct offences. Section 3 analyses offences where D requires *mens rea* as to her own future (T2) conduct, such as burglary; and section 4 analyses offences where D requires *mens rea* as to the future (T2) conduct of another party, such as complicity.

## 2. Present-Fault: Target and Definition Limits

*Mens rea* terms are designed, and can only be understood, in relation to their corresponding target.^14^ We cannot, for example, experience an abstracted state of ‘intention’ or ‘knowledge’ *simpliciter*. That would be illogical. Rather, when we speak of intentions or knowledge, we speak of intended *outcomes*, of known *facts*, and so on. Thus, when *mens rea* terms are defined for legal purposes, it is necessary to identify corresponding targets (or categories of target) to give those definitions meaning. Beyond intelligibility, correspondence is also necessary for *mens rea* terms to perform their normative role within an offence. Just as we cannot experience an abstracted state of ‘intention’, it would be equally illogical to blame D for so intending. *Mens rea* terms facilitate the criminal blaming of D because they provide an essential link between her agency and her wrongful conduct, as well as grading the culpability of that link across the different *mens rea* terms. Thus, for legal *mens rea* terms to function, they must do so in clear correspondence with the actions or events for which we are assessing D’s blame.^15^

**Figure 1:**
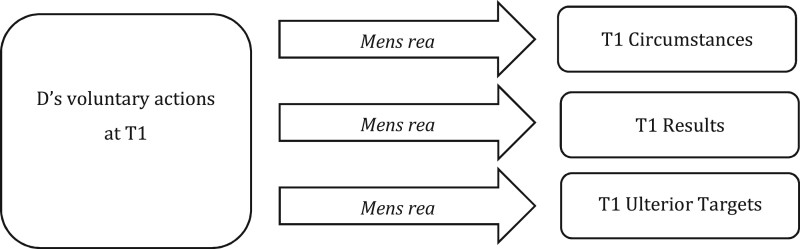
Present-conduct offences.

The importance of target elements for the definition of *mens rea* terms is recognised within the current law, but is limited to present-conduct offences. We illustrate the target elements of present-conduct offences in Figure 1.

The same *mens rea* term can be, and often is, defined differently as it applies to different T1 target elements. ‘Intention’ as to T1 circumstances, for example, is typically defined with a motivational requirement of purpose or knowledge (ie D must act now *because* of a fact, or with *knowledge* of it); and in relation to T1 results, it is typically defined with both a motivational and causal requirement (ie D must also act *in order to*, or with *knowledge that*, her action will bring the result about).^16^ The same structure of correspondence is applied to the definition of other *mens rea* terms as well, such as ‘recklessness’ (discussed earlier), ‘knowledge’, ‘belief’ and so on. These definitions do not make descriptive or normative sense without a clear corresponding target in mind; and just as importantly, the different targets (here, T1 circumstances and T1 results) necessitate different definitions of each *mens rea* term in order for that correspondence to work.

**Figure 2:**
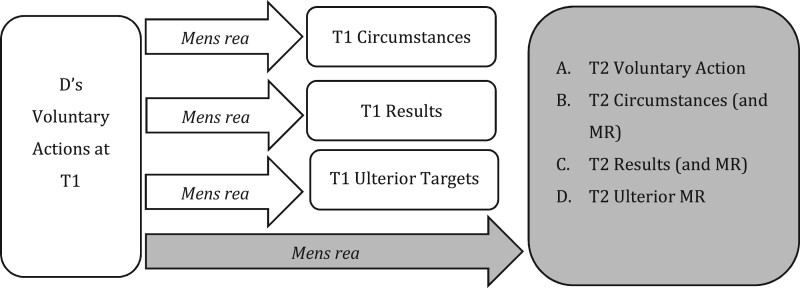
Future-conduct offences.

But this only takes us so far. When analysing future-conduct offences, as illustrated in Figure 2, new *T2 targets* must be considered. These new targets typically exist within D’s *mens rea* alone, though, in the case of complicity, they may require completed conduct.

The question is how *mens rea* terms apply to targets at T2? What does it mean within conspiracy, for example, for D (at T1) to ‘intend’ the elements of a future (T2) offence? The answer, crucially, is not clear. It is not clear because, although the definition of *mens rea* terms will often vary between the circumstance and result elements of D’s T1 actions, current criminal codes (and other legal sources) do not typically recognise a definitional distinction between *mens rea* as to T1 elements and T2 elements.^17^ Yet, a distinction is evidently required to make sense of such *mens rea* terms in practice.

Take the conspiracy example, where D must (at T1) ‘intend’ the elements of a future (T2) offence. As we have said, intention is currently defined in motivational (ie D acts because) or knowledge-based (ie D acts with knowledge that) constructions and, for results at least, is tied to D’s causal role in action. Yet none of these appear apt for explaining intention as to a future (T2) offence. At T1, D’s acts of agreeing the future offence may make that offence more likely to happen at T2, but, whether it is to be performed by D or another, it is difficult to claim that D’s acts at T1 are intended to *cause* any element of the future offence (ie D will acknowledge that further choices are to be made in action at T2, and it will be those acts that are intentionally causal). Equally, although D may plan the elements of a future offence, the agential aspects (ie T2 acts and results) cannot logically be *known* at T1 as a virtual certainty; and related T2 circumstances will also be more or less certain at T1.

Problems of this kind are rarely acknowledged by the courts, where *mens rea* terms are typically employed without specific definition. But in cases where definitions become important and contested, as we explore fully later, problems become clear. The principal authority on the *mens rea* of conspiracy, for example, provides as many conflicts and confusions as it does judicial opinions:^18^ doubting the potential for future focused ‘knowledge’ *tout court*;^19^ questioning if ‘belief’ can satisfy a ‘knowledge’ requirement;^20^ and even (in dissent) questioning whether a plan to continue ‘even if’ a criminal circumstance is present can be interpreted as a conditional intention.^21^ As Lord Hope correctly observes, ‘the concepts on which [the *mens rea* for conspiracy] were based are easy to state but not nearly so easy to apply in practice, [and have] been the subject of a vigorous debate ever since’.^22^ The same applies for other future-conduct offences. For example, efforts to understand *mens rea* for burglary and attempts became (at least at one time) contorted around distinguishing present (T1) and future (T2) settled intentions, prompting the Court of Appeal in *Husseyn* to comment that ‘it cannot be said that one who has it in mind to steal only if what he finds is worth stealing has a present intention to steal’.^23^ And within the jurisprudence of complicity, we have the Supreme Court in *Jogee* dismissing 30 years of *mens rea* interpretation as a ‘wrong turn’, before failing to articulate its preferred direction of travel for intending the future offence of another with any degree of clarity.^24^ These are not case- or offence-specific problems; they are systemic problems, and they require a systemic response.

## 3. Ulterior-Fault: Mens rea as to D’s Future Conduct

This section explores offences that require D’s conduct at T1 to be accompanied by *mens rea* as to future actions and/or offences by D herself at T2. The example of section 9(1)(a) burglary mentioned earlier, where D enters as a trespasser at T1 with intent to commit a listed offence at T2, is therefore apt. But examples beyond burglary are many and varied, from general inchoate offences such as attempt^25^ and conspiracy^26^ to a host of bespoke/substantive inchoate offences typically framed around possession with intent to perform future conduct,^27^ trespass/unauthorised access with intent to perform future conduct^28^ and so on.^29^

The important role played by offences of this kind, often marking the boundaries of criminalisation, has led to significant academic scrutiny.^30^ The predominant focus of such scrutiny is normative, debating the acceptable realms of criminal law. Our focus is different: we explore the analytical structures and definitions framing that debate and, in particular, the definition and analysis of *mens rea* applicable to T2 target elements.

We begin our analysis with D’s *mens rea* as to her own future T2 actions, before moving on to the circumstances and results of those actions.^31^

### A. D’s Mens rea as to D’s Future Actions: The Definitional Error

The question here is how D’s *mens rea* at T1 should be formulated or understood in relation to her future actions at T2. *Mens rea* as to *present* action is typically understood in terms of ‘voluntariness’, focusing on D’s volitional or non-involuntary movement.^32^ Thus, for burglary, D’s movements when trespassing at T1 must be voluntary. In whatever way ‘voluntariness’ is defined, however, the concept is clearly inapt to capture D’s *mens rea* at T1 as to future actions (eg T2 appropriation): D can perform a voluntary movement now, but she cannot be voluntary or volitional at T1 in performance of T2 movements she is not currently making.^33^ The current law avoids these confusions by asking if D ‘intends’ to act at T2, acknowledging the T1/T2 target distinction.^34^ However, this still leaves the challenge of defining ‘intention’ as to T2 action.

It is a challenge, we contend, where courts and legislators have come up short. There is no distinct definition of ‘intention as to D’s T2 actions’ currently recognised within the law. Therefore, by default, courts are left to apply definitions of intention developed within the present-fault paradigm (ie as to T1 circumstances and results) based on motivation, knowledge and causation in action.^35^ But these definitions do not fit their new target, creating conceptual and normative problems. Two examples of this can be seen in the discussion of ‘conditional intention’ and in the relationship between what is intended and D’s actions at T1.

Conditional intention is a contested concept, but essentially allows us to find criminal intent even where D’s purpose is contingent (eg D intends to Φ if x).^36^ Where D acts with intention as to a T1 circumstance or result, conditionality does not seem to play a role: D acts either *in order* to cause or with present *certainty*, with any previous conditions resolved in action.^37^ Definitions of intention are not therefore typically expressed in conditional form. But intention as to future T2 acts can certainly be conditional, and arguably will *always* be conditional: D knows at T1 that she will have a choice in action at T2.^38^ Thus, without separate definitions of intention for T1 and T2 targets, either *all* intentions become potentially conditional (problematic for intending T1 targets) or *no* intentions can be conditional (fatal for intending T2 targets).

The current law has struggled between these options, before settling on the first and least objectionable. The problem was revealed in a line of attempted theft and burglary cases in the 1970s,^39^ offences that require D to intend (at T1) to commit a future offence at T2. Where D’s plan was conditional (eg to steal *only if* the property was worth taking, or *only if* not disturbed), the courts were initially reluctant to find that it was intended; and they were surely right that, when acting at T1, D was not doing so *in order* to steal by this action.^40^ However, *not* finding intention on these facts renders such offences redundant, and was therefore unacceptable from a policy perspective.^41^ The line of cases therefore ended, predictably, with a lurch to the only option within the present-fault paradigm, accepting the potential for conditionality generally.^42^

The potential for conditional intention is now commonplace in English law,^43^ and is similarly presented within the US Model Penal Code as a general gloss to present-fault definitions of ‘purpose’ as to circumstances and results.^44^ The problem with this, however, as we have said, is that conditionality does not make conceptual sense when applied to T1 targets: D’s intention as to a T1 circumstance or result is necessarily complete in action at T1; there is no future point of agency to condition.^45^ Accepting conditionality as a general gloss to the meaning of intention therefore, although necessary for T2 targets, risks confusion and incoherence if applied to T1 targets. And this is exactly what we see in the seminal complicity case of *Jogee*,^46^ where, having rejected ‘recklessness’ as the *mens rea* for complicity, the UK Supreme Court explicitly endorsed conditionality as to D’s T1 intention to assist or encourage.^47^ The use of conditional intention makes little conceptual sense in this context; has been criticised by academics and international courts;^48^ and remains arguably the principal source of uncertainty for complicity liability.^49^

The second problem with using definitions of intention within the present-fault paradigm to target D’s acts at T2, is the conceptual relationship between D’s T1 actions and that target. This has gone largely unnoticed within the current law, but it is fundamental to our thesis, demonstrating that even with the gloss of conditionality present-fault definitions do not fit T2 targets. The problem here is that the relationship between T1 action and other T1 elements is structurally different from the relationship between T1 action and T2 action; and this relationship matters for the definition of intention. At T1, D does not ‘intend’ her future acts like a T1 result element of her present action: where D retains capacity,^50^ she knows that her T1 acts cannot *cause* her action at T2, and so it makes little sense to describe her T1 actions as being done with a purpose to bring about acts at T2 or foreseeing their causation as virtually certain.^51^ Equally, D does not ‘intend’ her future acts at T2 as she would a T1 circumstance element either: such future action is not an objective fact to be foreseen or desired any more than present action is; rather, it is an exercise of known agency. Simply put, even if we allow for conditionality, we still need to know what it means to ‘intend’ T2 action and this cannot be provided within the present-fault paradigm.

The solution, we contend, is to identify a new definition of ‘intention as to one’s own future action’ (ie in addition to current definitions as to T1 circumstances and results). Such a definition must include the potential for conditionality, a potential that can then be explicitly excluded from intention as to T1 targets; and it must also explain the necessary connection between D’s T1 actions and intended T2 actions. We have suggested elsewhere that a ‘commitment model’ should be employed: ‘D intends (at T1) to act at T2 where she commits to doing so under certain conditions’.^52^

**Figure 3:**
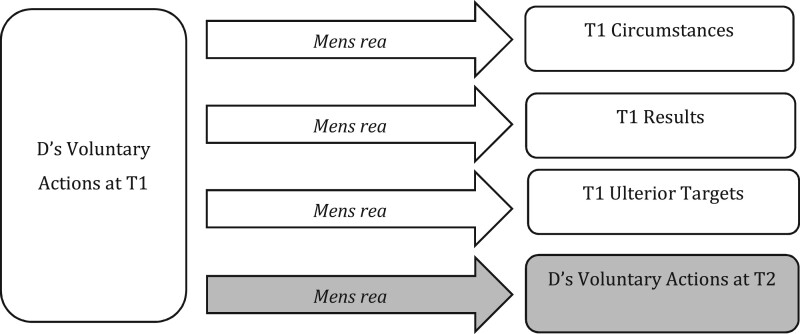
MR as to D’s actions at T2.

The idea of conditional commitment as a species of intention throws up several important normative definitional questions, relating to D’s attitude or endorsement of the condition,^53^ D’s perception of likelihood,^54^ whether subjective conditions are included^55^ and so on. We may also question the use of ‘commitment’ as appropriate for D’s T1 decision making, in terms of both the link this provides to T2 action and what it might require of D at T1 (eg in acting to prepare for T2, or at least not acting in opposition). We do not seek to debate or resolve these issues here, though none should be seen as unduly problematic.^56^ Rather, our point here is that a new formulation of ‘intention’ of the sort just outlined is required in order both to allow us to make sense, functionally, of ‘intention’ in law as to D’s future action at T2 and to bring to the fore associated normative definitional issues for discussion.^57^ The definition of *mens rea* as to D’s T2 action becomes a separate point of concern alongside (and not amalgamated within) *mens rea* as to T1 targets, illustrated in Figure 3.

### B. D’s Mens rea as to D’s Future Offence: The Analytical Error

Section 3A focused on D’s *mens rea* as to her future actions alone, but most of the offences under discussion do not simply require *mens rea* for that target. Where D’s offence includes ulterior *mens rea* as to a future ‘crime’, such as section 9(1)(a) burglary, we must also consider D’s *mens rea* at T1 as to the circumstances and results of her T2 action. At T1, when entering as a trespasser, for example, we ask if D is ‘intending’ her T2 action to result in GBH or is ‘intending’ or ‘knowing’ that her T2 act will dishonestly deprive another of their property, and so on.

On its face, this appears to raise a new definitional challenge akin to that discussed in section 3A, requiring us to ask how present-fault *mens rea* terms might be redefined to engage T2 targets. And interestingly, as we will explore, this *is* the challenge highlighted by appellate courts and commentators in this area.^58^ However, we contend that this is misconceived. Rather than presenting extended T2 elements as unique targets to be understood entirely from a point in action at T1, we explore how the anticipated point of action at T2 should factor within our analysis. We refer to the failure to recognise this as the ‘analytical error’. Once corrected, present-fault definitions of *mens rea* terms can be reemployed, understood as part of the wider event D has committed to perform *at T2*.

#### (i) Efforts at definitional adjustment?

To understand the importance of this analytical error, it is essential first to understand how and why it might be mischaracterised as definitional. This involves examining why courts and commentators have redefined or avoided terms such as ‘intention’ or ‘knowledge’ when applied at T1 to circumstances and results of a future T2 action.

The apparent definitional challenge can be understood as follows. If D does not cause her future T2 actions by her actions at T1, then neither does she (at T1) cause any results flowing from those future actions. Thus, D cannot act at T1 with an ‘intention’ (as currently understood) as to the results of her actions at T2. Equally, if ‘knowledge’ as to a circumstance requires a *true* belief in its existence, then D will often lack (and arguably will *always* lack) knowledge at T1 as to circumstances at T2 that are yet to exist, may change or rely on unpredictable interventions.^59^ Beyond ‘intention’ and ‘knowledge’, similar definitional challenges are apparent for all *mens rea* terms that require an *objective* assessment of D’s context in acting.^60^ In each case, if such terms are applied to T2 circumstances and results, these objective facts may not be known or knowable at T1. Such problems are variously recognised and not generally disputed, and all acknowledge that their resolution is far from easy.

Perhaps the best example to illustrate the (apparent) definitional challenge, and the nature of current legal responses, is conspiracy.^61^ The statutory definition of conspiracy contains an express requirement that at the point of agreement (T1) D must ‘intend or know’ any circumstances required for the agreed future principal offence to be committed at T2.^62^ In a series of cases concerning conspiracy to launder money, courts have been forced to grapple with the requirement that D (at T1) need not simply have ‘suspicion’ concerning the criminal origin of property (sufficient where the laundering offence is completed), but must ‘intend or know’ that criminal origin.^63^ This seems to engage the definitional challenge: at T1, D often cannot intend or know (as currently defined) a circumstance of her future actions at T2.

The challenge eventually made its way to the House of Lords in *Saik*,^64^ which remains the leading authority on the *mens rea* of conspiracy. Saik was initially convicted of conspiracy to launder the proceeds of crime based on his clear *suspicions* that the money was ‘hot’, a conviction upheld in the Court of Appeal but quashed in the House of Lords as insufficient for intention or knowledge. Central to the case, then, was what it means to ‘intend’ or ‘know’ at T1 the circumstance of a T2 offence. In answering this question, the Lords drew liberally from an article by Ormerod (published after the Court of Appeal decision) that provides a thorough investigation of the various ways knowledge and intention could be defined to assist the court.^65^ Ormerod contended that ‘neither word is ideally suited’ in its current form,^66^ and that the best way forward would be to use a version of ‘conditional intention’: D intends or knows the future circumstance at T1 if she agrees (at T1) to ‘continue even if that [ie the future circumstance] may be the case’ and the circumstance does materialise in fact.^67^ This formulation was accepted in Baroness Hale’s dissenting judgment, but rejected by the majority as a potential ‘watering down’ of the knowledge/intention requirement.^68^ We return to this conditional intention option later, but for now it is useful to highlight the uncertain nature of the concepts being protected from dilution.

Recognising the same definitional problems as Ormerod (ie with attaching present-fault definitions to T2 targets), the majority judgments are only able to apply requirements of ‘knowledge’ and ‘intention’ by effectively and inconsistently remoulding their definitions.^69^ The clearest example of this comes from Lord Brown (concurring with Lord Hope), who stated that ‘if an agreement is made to handle goods *believed* to be stolen’,^70^ he would have little difficulty concluding ‘the conspirators intended or knew that they would be stolen’.^71^ Treating subjective belief as ‘knowledge’ is a considerable definitional shift, to say the least. Lord Nicholls (with whom Lord Steyn agreed) did not endorse this interpretation, but presented an equally unattractive alternative. This involved drawing a new distinction between laundering property ‘identified’ at the time of the conspiracy, where only a narrow knowledge requirement (involving first-hand experience) would suffice,^72^ and ‘unidentified’ property, which would require intention.^73^ It is a factual distinction that may not always be clear, and still leaves the term ‘intention’ undefined in the latter scenario.

Confusions surrounding the definition of intention and knowledge following *Saik*, and concerns that D’s acquittal was normatively unsatisfactory led to a Law Commission referral to consider both attempts and conspiracy.^74^ The Commission recommended an alternative *mens rea* of recklessness, seeking thereby to avoid (rather than resolve) the apparent definitional problems with intention and knowledge as to T2 targets.^75^ For the Commission, a knowledge standard was not only problematic for its requirement of ‘true’ belief in a future fact, but also because of the restrictive degree of foresight necessary at T1. The Commission’s conspiracy to rape example illustrates this point:

D1 and D2 agree to have sexual intercourse with V [the victim] whether or not V consents. We consider that this ought to be regarded as a conspiracy to commit rape but, following *Saik*, it will not necessarily be so regarded … the prosecution has to prove that D1 and D2 knew at the time of the agreement that V would not consent. However, it would be very easy for them plausibly to claim that they merely thought V ‘might not’ or ‘probably would not’ consent.^76^

The Commission observed an equivalent problem within the law of attempts, proposing the same move to recklessness.^77^

#### (ii) What is wrong with the ‘definitional’ approach? Understanding the analytical alternative

*Saik* and the Law Commission’s examination of the issues arising from that case serve to illustrate two overarching points. First, they illustrate how difficult it is to accommodate present-fault vocabulary and definitions within offences requiring two conduct events, where *mens rea* must exist at T1 in relation to results and circumstances of action at T2. Secondly, however, we would argue that the efforts at resolving the problem have not been successful because the definitional debates just explained are constructed upon a core analytical error. The error here, grounded within the present-fault paradigm, is the analysis of offences with multiple agential events as if there was only one relevant point of coincidence (ie D’s actions at T1 alone).

**Figure 4:**
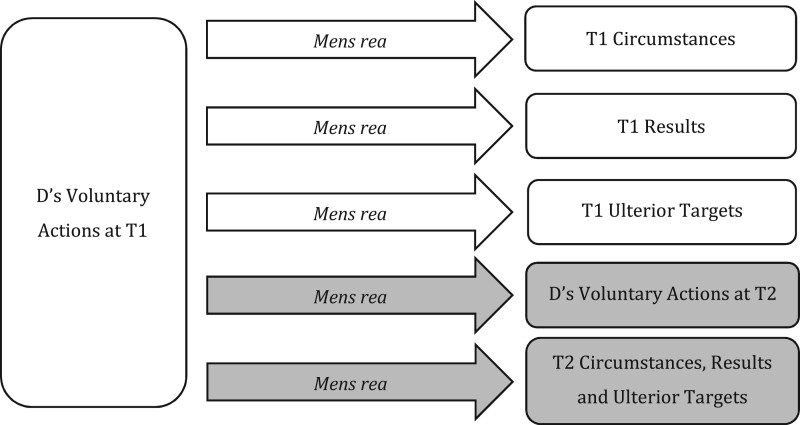
D’s MR as to T2 elements (analytical error).

**Figure 5:**
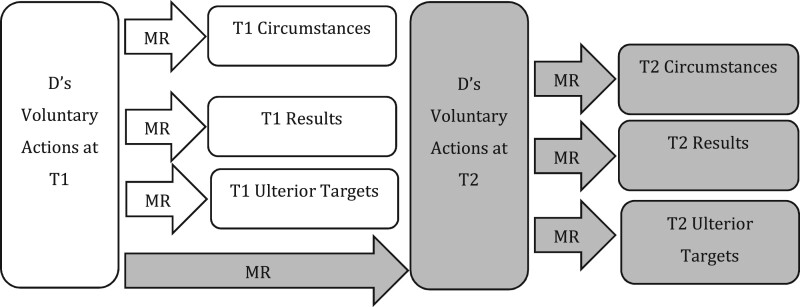
D’s MR as to T2 elements (preferred approach).

On one level, of course, it is correct to highlight a single core point of coincidence at T1. It must be possible, at T1, to demonstrate the coincidence of all *mens rea*, including ulterior *mens rea*. For example, D’s intention to steal from a house at T2 must be present at T1, when entering that house as a trespasser, for D to commit section 9(1)(a) burglary.^78^ However, there is a crucial difference between requiring ulterior *mens rea* at T1 (clearly true) and defining ulterior *mens rea* as to T2 targets in relation to (i) D’s action at T1 as opposed to (ii) *D’s intended action at T2*. Approach (i), we contend, is the analytical error, whereas (ii) reflects the correct approach analytically. The different approaches are represented across Figures 4 and 5, with our preferred approach in Figure 5.

Figure 4 sets out an analysis of *mens rea* for T2 circumstances and results that ignores T2 actions, working from a point of coincidence at T1 and defining *mens rea* terms so they can apply to future events from this point. We contend this *creates* the definitional challenges discussed above since it forces us to ask what D ‘knows’ at T1 about future events at T2; what D ‘intends’ to cause at T2 by her actions at T1; and so on.

We contend that it is more appropriate, both conceptually and analytically, to address *mens rea* as to T2 targets in relation to D’s T2 actions as a second point of coincidence, as represented in Figure 5. Rather than looking for actualised *mens rea* states at T1 (ie present knowledge or intention), the focus can shift to what D has committed to (ie the detail of the T2 event). For example, where D commits at T1 to taking another’s property at T2 (the future actions required for section 9(1)(a) burglary), the question becomes whether she commits to doing so in anticipated *T2* circumstances that we would consider dishonest, and with an intention *at T2* to permanently deprive. The problem here is not in defining the target of D’s *mens rea*;^79^ it is about where and how such *mens rea* must coincide with D’s actions (ie actions at T1 and intended actions at T2).

Taking proper account of the relationship between T1 and T2 outside of the present-fault paradigm provides several pay-offs. Crucially, working from D’s intended acts at T2, present *mens rea* definitions can be applied to T2 targets.^80^ The apparent definitional error, across all *mens rea* terms that include an objective element, is eliminated. And beyond this, it also provides a normatively preferable mechanism for constructing and applying inchoate offences that include multiple points of agency.

The Law Commission example of conspiracy to rape, quoted above, provides a useful illustration. Criticising the precedent from *Saik*, the Commission correctly observe that requiring D *at T1* to know that V will not consent *at T2* (as well as being definitionally problematic) is unduly restrictive of liability.^81^ However, because it still defines T2 *mens rea* in relation to T1 action (ie the analytical error), the Commission’s choices for addressing this normative problem are constrained. In order to expand liability, it acknowledges two main options. The first, which it recommends, is to expand *mens rea* for conspiracy to include potential recklessness *at T1* as to circumstances *at T2*. Recklessness is less obviously problematic than knowledge when applied to future events,^82^ and would certainly expand liability. However, the recommendation has not been implemented, and may be criticised for its potential to overextend conspiracy liability^83^ and create a problematic distinction between circumstances and results.^84^ The second option, which the Commission rejects, is equivalent to the conditional intention approach recommended by Ormerod and endorsed by Lady Hale in *Saik*. This approach promises liability in cases such as the conspiracy to rape hypothetical, on the basis that if D intends penetration foreseeing a potential lack of consent, then he intends *both* consensual and non-consensual penetration in the alternative.^85^ The problem with this approach, however, recognised by the Commission and the majority in *Saik*, is that it defines conditional intention in a way that collapses into foresight/recklessness;^86^ and, in some cases, it relies on knowing the truth of T2 circumstances at T1, which cannot be guaranteed.^87^

The better approach, we argue, is to maintain a requirement of intention or knowledge as to T2 circumstances, but to analyse this requirement in relation to D’s actions at T2. This builds upon our analysis of intention as to T2 acts in the previous section. Thus, in the conspiracy to rape hypothetical, our focus should be on the content of D’s commitment at T1 as to future action (ie the act of penetration) at T2: if D intends (T1) to act even if he comes to know before T2 that V does not consent, then he commits to acting with the relevant intention or knowledge as to the T2 circumstance. Analysing T2 *mens rea* in this way means that a state of knowledge as to future facts is not required at T1, and the potential for conspiracy liability is expanded.

This approach does not *simply* expand liability, however, and does not lead us into the same problems as the Commission’s recommendations. Rather, shifting focus to explore what (at T1) D has committed to at T2, provides a better mechanism for identifying relevant culpability. *Even if* we could define T1 *mens rea* terms to apply to T2 events, there are several normatively unimportant reasons why D may not intend or know a future circumstance at this earlier point. For example, D may know that the circumstance has not arisen at T1,^88^ that it may change,^89^ that it relies on her own future conduct^90^ and so on; or D may simply be uncertain at T1. But a lack of knowledge or intention of this kind at T1 tells us little about D’s culpability as to an intended and/or agreed offence at T2.^91^ What ought to matter, we contend, is whether D has committed to completing the offence at T2 with such intention or knowledge *at that point*. This form of analysis applies not only to conspiracy, but to all offences where D requires *mens rea* as to their own future conduct.^92^

Almost all of the commentators critiqued in this section have come close to our preferred conclusions. Most notably, both Ormerod and Hale provide illustrations of their approach that include T1 commitment to actions at T2 *even if* relevant knowledge is gained.^93^ However, the reason their approach has been easy for some to dismiss^94^ is because they also include examples where D does not anticipate knowing or intending when acting at T2.^95^ Analysing these latter examples as ‘intentional’, because D intends to act regardless of circumstances that will remain unknown, is to collapse intention into foresight/recklessness. The Law Commission too, when discussing conditional agreements in conspiracy, has recognised the potential for liability where elements of a ‘*hypothetical* course of conduct’ (ie one conditionally intended) includes elements that will only be known or intended at T2.^96^ This recognition is presented as a limited exception in cases of explicit conditionality. But in acknowledging the difficulty, and allowing an approach that focuses on anticipated intention and knowledge at T2 (ie our preferred approach), the Commission is attempting to sidestep a much greater issue. All future conduct is hypothetical at T1; these problems cannot be limited to explicitly conditional agreements alone; so, the approach we suggest must be generalised.

The analytical error discussed here may have persisted for so long because of the unfortunate tendency for conspiracy to be charged for completed offences.^97^ Where the principal offence is completed, it is tempting to take account of certain T2 *facts* (circumstances and results) that have materialised. However, in the case of the offences under discussion, analysis should only focus on D’s intentions and foresight of these elements as *incomplete* at T1. Another explanation for the current approach is that it appears to maintain high thresholds of *mens rea*. Where D lacks present-fault knowledge or intention at T1 as to the details of a future event at T2, but rather commits to a *potential* of acting with such knowledge or intention, some may worry that D’s commitment is culpably insufficient. Like the hypothetical journey where D agrees to speed *if necessary*,^98^ we may want more in the form of likelihood or endorsement of that possibility from D before we find liability. If this is correct, we contend that the issue for debate lies in the definition of intention as to future *action*,^99^ ie the definition of D’s T1 commitment, where requirements of this kind could be included without the collateral confusion and incoherence discussed here in relation to circumstances and results.

## *4*. Ulterior-Fault: Future Conduct of Another

In this section, we examine future-conduct offences where D’s action at T1 is accompanied by *mens rea* as to conduct by *someone else* (P) at T2. Several offences present this possibility. Complicity is perhaps the most obvious, but the same possibilities are presented by conspiracy (where D agrees with P that P will commit the principal offence) and the inchoate offences of assisting and encouraging.^100^ The issue is how we understand D’s intention (or other *mens rea*) at T1 as to the future conduct of P at T2 (including P’s acts, circumstances and results) and P’s *mens rea* at T2.

It is important at the outset to emphasise why we have identified D’s *mens rea* as to conduct *by another* as requiring specific and distinct examination. It is because there is a categorical difference between crimes which require D to have *mens rea* at T1 as to the future action of *someone else* as distinct from *themselves*. Where D ‘intends’ (etc) the future conduct of another, not only is T1 (again) a step removed from the conduct of the principal offence at T2, but, significantly, the T2 step is now not controlled by D. Whatever *mens rea* we demand of D at T1 in respect of P’s conduct and *mens rea* at T2, it must be configured in light of the fact that D has no agency at T2—it is out of their hands, lying entirely with P.

Although D’s lack of anticipated agency at T2 is therefore crucial, it is remarkable that when the courts have (rarely) acknowledged a difference between present-fault and future-fault definitions of *mens rea* terms, they have never identified the question of whether T2 conduct is completed by D or another as having any significance. This is reflected, for example, in the discussion and hypotheticals used by the Supreme Court when analysing the meaning of ‘intention’ for complicity in *Jogee.*^101^ So too with conspiracy. For example, although one of the most highly criticised House of Lords authorities on the *mens rea* of conspiracy, *Anderson*,^102^ is notable for focusing on D’s *mens rea* as to another committing the agreed offence, this feature is left largely unremarked upon or unrecognised within textbooks and commentaries. The same is true within the Law Commission’s most recent consultation^103^ and report^104^ on conspiracy, where, despite explicit criticism of *Anderson*, just four boxed examples (from over 50) involved D who was not herself planning to commit the principal offence, and none of these four were used to explore *mens rea* questions.

Several problems flow from, and/or are exacerbated by, the failure to recognise and account for conceptual distinctions between *mens rea* as to D’s own future conduct and that of another. It stops us investigating cases like *Anderson* for the unique challenges they present separate from conspiracy cases where D plans to commit the T2 offence herself; and it deceives us into thinking a single approach can be used in both contexts, as is reflected in both the current law and existing reform proposals. It is no surprise, therefore, that offences requiring *mens rea* as to the future conduct of another, such as complicity, remain deeply contested as to their *mens rea* requirements, presenting some of the most polarised and intractable debates about the definition and application of *mens rea* terms.

Consider disputes between commentators over complicity liability,^105^ and in particular what it means for D to ‘know the essential elements of the principal offence’.^106^ For Ormerod^107^ and others,^108^ because they see it as conceptually impossible to ‘know’ future elements, the term must be interpreted as requiring a form of ‘intention’. However, for Simester^109^ and others,^110^ D’s lack of causal agency at T2 makes ‘intention’ conceptually impossible, and ‘knowledge’ must instead be interpreted as something more like a settled belief. Fundamental definitional conflicts of this kind remain unresolved despite a succession of House of Lords/Supreme Court interventions,^111^ alongside periodic Law Commission projects.^112^ It is only by resolving these conceptual disputes that we can provide the tools required for constructive normative debate, and (again) we contend that the key to such disputes lies beyond the present-fault paradigm.

As in section 3, our analysis begins by identifying a definitional error in what it means to intend at T1 *future actions* at T2, this time the actions of P. Our focus then moves to the associated elements of P’s T2 offence, including its circumstances, results and *mens rea*.

### A. D’s Mens rea as to P’s Future Action: The Definitional Error

We begin with the definition of D’s ‘intention’ as to the T2 actions of P. Unlike D’s *mens rea* as to her own future actions, other *mens rea* terms are sometimes applied here.^113^ But it is useful to focus on intention only, as the most common and most problematic standard.^114^ Such intention is required for conspiracy, where the parties conspire for P to commit the future offence;^115^ where D assists or encourages P for the purposes of a section 44 SCA offence;^116^ and, arguably, following the dominant interpretation of complicity.^117^

As with intention relating to D’s own future actions, we contend that a new and distinct definition of ‘intention as to the future actions of another’ is required.

#### (i) Definitional possibilities within the present-fault paradigm

The definitional error, as we term it, involves applying (or attempting to apply) present-fault definitions of intention to the new T2 target. We start our discussion here again, as we did in section 3A, because current law and commentary seem to adopt this approach. But also, the fact that P’s actions at T2 are not controlled by D’s agency makes present-fault definitions of intention (at least superficially) more attractive than when applied as to D’s own T2 actions.

The most common approach/analysis currently adopted is to interpret D’s ‘intention’ as to P’s future actions as an *intended result*: D acts at T1 in order to cause P’s actions at T2 or foreseeing them as a virtually certain consequence of her T1 actions. This interpretation is preferred, for example, in most Law Commission publications, in their analysis of current law as well as reform proposals.^118^ Even Robinson, who distinguishes D’s own ‘future-conduct intention’ for special treatment, concludes that intending the future conduct of another ‘does not appear to present a culpability requirement different from … “future result culpability”’.^119^

The problem with this analysis is that acting *in order to cause* and/or foreseeing a *causal inevitability* is only conceptually possible if we can establish a causal link between D’s actions (T1) and P’s actions (T2). And the law has been emphatic in rejecting legal causation in these circumstances, highlighting P’s free and informed choice in action as breaking any causal chain.^120^ This has led some commentators to conclude that ‘intention’ is simply not conceptually viable in such cases;^121^ and, just as revealing, this has led others towards amended definitions of present-fault intention.^122^ For example, although Robinson claims that D can intend P’s acts in the same way as T1 results, he defines this as ‘culpability as to causing *or assisting* the resulting *offence*’.^123^ The problem here is that an intention to assist (as opposed to cause) is not reflected in the US MPC definitions of *mens rea* as to results; and so (*contra* Robinson) its inclusion in his discussion becomes a marker of conceptual difference.

The alternative, still within the present-fault paradigm, is to analyse D’s intention as to P’s future actions as an *intended circumstance* of T1 action: D acts at T1 with the hope that P will act and/or knowing that P will act in a particular way at T2. Although Simester rejects intention as to P’s conduct *tout court* in the context of complicity, he (and others) accepts a role for this form of intention in conspiracy cases where D and P agree that P will commit an offence.^124^ Interpreting intention as to P’s actions in this way avoids the causal challenges just discussed, as hoped for or known circumstances do not necessarily assume a causal connection from D’s actions at T1. But conceptual and normative mismatches arise quickly. As future events, the use of a ‘knowledge’ standard draws us back into debates about conceptualising knowing the future;^125^ and even where this is interpreted as a settled belief, the need to accommodate P’s T2 agency makes a high threshold (akin to a virtual certainty) much more difficult to maintain.^126^

More broadly, the cost of the ‘intention as to circumstances’ approach is a definition of intention that seems to miss an essential element of D’s culpability: where D’s actions are motivated to increase the likelihood of P’s offence, this seems like an appropriate target for blame; and where it is not so motivated, criminal blame may be inappropriate. This is at issue, for example, when debating the relative culpability of shopkeepers (and others) who, in the course of their employment, provide assistance to known offenders.^127^ Thus, moving to a definition of intention or knowledge that fails to capture this dimension of culpability seems inappropriate.

#### (ii) Correcting the definitional error

Approaches rooted in the present-fault paradigm do not provide viable solutions. Treating P’s T2 action as an intended result of D’s T1 action is conceptually incoherent; and treating it as an intended or known circumstance fails to capture and express D’s culpability. Thus, our attention turns to other possibilities, outside typical present-fault paradigmatic approaches.

One option would be to re-employ our preferred definition of ‘intention as to D’s own future actions’—a conditional commitment at T1 to decide to act at T2^128^—extended to cover intention as to the future actions of another. This has obvious attractions. We have defined such intention to target T2 action; consistency here would prevent added complexity in conspiracy cases;^129^ and the potential for conditionality has already been endorsed by the Supreme Court in *Jogee*.^130^

However, it should be apparent that this approach cannot work. Where D intends the future actions of another, D knows that this action (P’s action) is not something D can pre-emptively commit to: D cannot commit to a future decision to act because the future decision will not be hers to make. This rules out a future commitment model for intention, and (*contra* extensive *obiter* in *Jogee*) it also rules out conditionality. Where D has only a single point of agency at T1, although conditions may have preceded her decision *to act then*, any decisions D makes in action (ie intentions) are complete/non-conditional at this stage.^131^ D may seek to influence P’s future actions to bring about variously general and/or quite specific future outcomes; D may have multiple intentions simultaneously, recognising various contingent facts; but to label this ‘conditional intention’ is to imply a level of agency at T2 that D lacks.^132^

**Figure 6:**
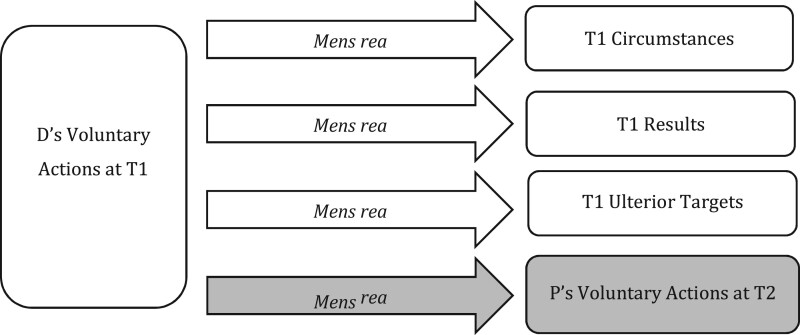
MR as to P’s actions at T2.

We contend that this apparent definitional impasse ought to be resolved by formulating a new definition of ‘intention as to the future actions of another’. As with intention as to D’s own future actions, this requires us to recognise P’s T2 actions as a distinct target outside (and not amalgamated within) other T1 targets. We illustrate this in Figure 6.

How should we define D’s T1 ‘intention’ as to P’s T2 actions? By way of illustration,^133^ we suggest the following: D intends the acts of P where she acts in order to cause assistance or encouragement of P’s acts or foresees such effects as a virtual certainty. This approach maintains a distinction between *mens rea* as to D’s T1 circumstances and results (present-fault targets) and P’s T2 actions (future-fault target). D’s T1 intention as to P’s T2 actions—intention to cause assistance or encouragement—targets things D can control, affect and therefore intend at T1.^134^

There are two main advantages to this approach. First, it allows engagement with D’s causal ambitions as a target for blame, but does so in a way that recognises the unique causal relationship existing between knowing agents.^135^ D cannot cause another’s acts as she might cause a natural event, but she can assist or encourage (she can create or endorse reasons for P to act in a certain way), and this can be reflected within a causal model of intention. Secondly, and just as importantly, a distinct definition of intention as to the actions of another prevents definitional choices here inappropriately affecting the definition of intention elsewhere. Our understanding of intention as to T1 results, in particular, is not subverted to make liability possible in these cases;^136^ and more positively, conceptual space is created for an appropriate normative debate about how intention as to another’s actions *should* be defined.^137^

### B. D’s Mens rea as to P’s Future Offence: The Analytical Error

**Figure 7:**
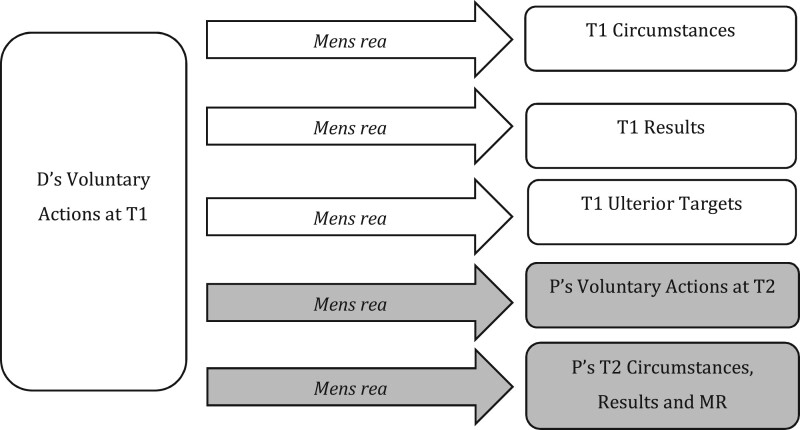
D’s MR as to T2 elements (analytical error).

In this final section, our focus shifts again to the wider elements (ie beyond action) at T2, exploring D’s T1 *mens rea* as to the T2 circumstances, results and *mens rea* required for P’s offence. This raises many of the same debates and contortions we encountered in section 3B when considering the position regarding the circumstances and results of D’s own actions at T2. Here we identify a similar analytical error, and demonstrate how many current legal problems can be avoided by correcting this error. As indicated in Figure 7, this arises where courts and commentators analyse D’s *mens rea* as to T2 elements as if T1 was the sole point of coincidence at issue.

**Figure 8:**
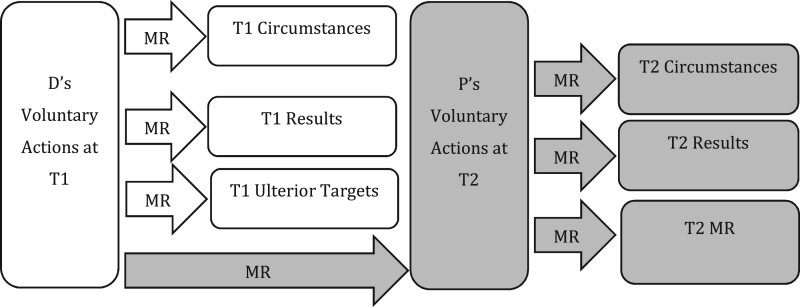
D’s MR as to T2 elements (preferred approach).

The solution we offer, illustrated in Figure 8, is in the same vein as that argued for in section 3B, though adjusted to take account of the fact that we are here dealing with the conduct and *mens rea* of P at T2. Thus, we contend that D’s T1 *mens rea* as to T2 elements should be analysed to take account of a second point of coincidence at T2, including the potential to find intention/knowledge/foresight as to P’s T2 circumstances, results and *mens rea* on the basis of D’s T1 intention that P should act *at T2* with such intention/knowledge/foresight.

In the brief subsections below, the offences of conspiracy and complicity are discussed. We highlight examples of the analytical error, and explain how each may be better analysed within our framework.

#### (i) Conspiracy

Where D and P conspire to complete a principal offence at T2, and this offence is to be committed by P alone, D (like P) must intend each element of P’s offence.^138^ So, beyond P’s actions (discussed above), what does it mean for D to intend P’s future circumstances, results and *mens rea*?

Current analysis of such *mens rea* provides a paradigm example of the analytical error. Rather than analysing D’s intentions as to the T2 event (ie what D commits to do at T2, or what D intends of P at T2), courts and commentators have assumed that all *mens rea* as to T2 events must be present at T1. This leads to misplaced debates about the current law, such as whether it is possible for D to ‘know’ a T2 circumstance at T1, as well as misplaced reform proposals, such as replacing a T1 knowledge requirement with recklessness.^139^

We contend such discussion is misplaced because, whether the plan is for D or P to commit the offence at T2, D’s *mens rea* should be analysed to take account of T2 as a separate point of coincidence. Thus, for example, where D and P agree that P will have sex with V at T2, it should be irrelevant whether D intends or knows *at T1* whether V will consent.^140^ Rather, focusing on the detail of the T2 event, we should ask whether D intends P to proceed with an act of penetration even if it becomes clear to P *at T2* that V is not consenting. If D’s intention encompasses an event of this kind, where V is intentionally raped by P, then D satisfies (on our analysis) the *mens rea* for conspiracy to rape; and this should be clear regardless of D’s beliefs at T1 regarding V’s likelihood of consenting.^141^

#### (ii) Complicity

Complicity is a more complicated case, not least because of current uncertainty about what *mens rea* D requires as to P’s principal offence. For the sake of discussion, we have interpreted the current law as requiring intention as to P’s future actions (discussed above) and *mens rea*, and knowledge as to associated circumstances and results.^142^

Echoing the analytical errors discussed for conspiracy, the requirement that D should ‘know’ the essential elements of P’s offence has often led to an exclusive and problematic focus on T1. We see this, for example, in debates about whether it is possible (at T1) to ‘know’ the future elements of P’s offence,^143^ and we see it in the Supreme Court’s mishandling of conditional intention in *Jogee*.^144^ In each case, problems arise because courts and commentators seek to apply causal and/or objective elements at T1 when the relevant point of agency (as to T2 circumstances and results) is exercised by P at T2.

The approach we developed in section 3B, and applied to the analysis of conspiracy above, takes us a considerable distance in correcting the analytical error here as well. For this approach, as long as D intends at T1 that P will act at T2 with intention or knowledge as to the elements of the principal offence *at that time*, we may fairly attribute the same intention or knowledge to D. The case of *Maxwell* provides a useful illustration, in which D led P to a particular location but was uncertain which offence P might commit on arrival.^145^ The court usefully acknowledges that D may have a ‘list’ of potential offences in mind in a case of this kind, and that as long as one of these is completed by P, then liability is appropriate. D does not have knowledge (or belief) as to any one offence at T1, but he must intend to assist or encourage a range of potential acts that involve P knowingly committing an offence at T2.^146^

Complicity, unlike conspiracy, however, is not limited to cases where D and P *both* intend or know the elements of the T2 offence. For example, D may intend (at T1) that P will act at T2 whilst P is reckless as to an offence element (eg reckless as to V’s consent for sexual penetration). Employing our preferred analytical approach, we might simply take from this that D is also reckless as to the relevant element and should not therefore (under the current law) be liable as an accomplice. But what if D holds a belief at T1 that V will not consent to P at T2, independent of D’s perception of P’s future beliefs? The Law Commission engaged with examples of this kind in their most recent report on complicity, recommending that D should be liable for complicity where she holds such a T1 belief.^147^ We agree. Note, however, that this approach does not revert to the analytical error: D’s belief is not *present*, but *future* focused, concerned with the circumstance *as it might exist at T2*; and note also the choice of ‘belief’, a *mens rea* standard that does not include an objective limb and does not assume causation.

## 5. Conclusion

Much of the material within this article is inevitably complex, discussing offences constructed around multiple conduct events, with both T1 and T2 *mens rea* targets; and within each relevant offence we are confronted with seemingly intractable legal puzzles, with varying judicial and academic responses. It is easy to become lost within such puzzles. But it is also important to recognise the value of engaging with complexity where this is necessary to understand and apply the criminal law fairly. Our thesis identifies and engages with complexities that are otherwise obscured in the common law.

The category of future-conduct offences is large and increasing, often marking the boundaries of criminalisation, and the general part problems we identify are fundamental to understanding and applying them. Without a clear conception of the general part issues we identify, definitional and reform debates within and across special part and general part offences are undermined. The structural problems we have explored, common across this category of offences, are too important to ignore.

Whether focused on the future conduct of D (section 2) or the future conduct of another (section 3), our discussion has sought to identify and challenge two core legal errors. The first—‘definitional error’—is identified where current present-fault definitions of intention (as to T1 circumstances or results) are applied to future actions. We contend that such definitions cannot and should not be applied, and that attempts to do so have created confusion both in the understanding of future-conduct offences and within those present-fault definitions themselves (ie where definitions are morphed in an attempt to fit their new context). New definitions of intention as to (i) D’s own future action and (ii) the future action of another are therefore essential additions to the definitional general part. The second core legal error—‘analytical error’—is identified where the broader conduct and *mens rea* elements of the T2 offence are analysed from T1 alone, without apparent recognition of T2 as a distinct point of coincidence. The error here is perhaps more subtle than the first, but arguably more damaging to the application of these offences. Focusing on completed *mens rea* states at T1 creates definitional mismatches and fails to target relevant points of culpability. This is in contrast to our approach, which demonstrates how present-fault *mens rea* definitions can be applied with reference to intended actions at T2.^148^

Our analysis, beyond the present-fault paradigm, has clear and profound implications for the understanding and application of *mens rea* terms. But it also has implications beyond this, which we have not had space to engage with here. Two prominent examples will suffice. The first relates to the *actus reus* elements of multi-conduct offences, and our understanding of ‘causation’ and ‘supervening events’ in the context of complicity in particular. As with causal understandings of ‘intention’, courts and commentators are correct to conclude that the definition of ‘causation’ within the present-fault paradigm cannot be applied to the actions of a secondary party,^149^ a simple observation that is used to dismiss so-called ‘causal accounts’. However, if we accept that causation as to another’s actions may be conceived differently outside of the present-fault context, as we did in our section 3A discussion of intention, then conceptual space is created for Gardner and others who have argued for a form of indirect-causation.^150^ The second example, demonstrating the broader implications of our framework, lies in prior-fault rules such as intoxication. These rules are currently disguised and distorted within a defence-based analysis that warrants no repetition here.^151^ But, in substance, these are rules that criminalise conduct at T1 (voluntary intoxication) that results in harms at T2 (the *actus reus* of a basic intent offence).^152^

In these examples, as with the core *mens rea* focus of this article, the challenge is to rationalise criminal law concepts that bring together multiple conduct events. It is a challenge we have engaged with here, and one that requires us to push the paradigmatic boundaries of the general part.

## Footnotes

1 We briefly return to the wider implications of our thesis, beyond *mens rea*, in the conclusion, but limiting analysis to *mens rea* provides necessary focus for the central part of the article.

2 eg whether ‘malicious’ does or should represent a separate state of culpability from ‘recklessness’, etc.

3 eg the consolidation of *mens rea* terms within the US Model Penal Code, and similar attempts within English law. See Kenneth Simons, ‘Rethinking Mental States’ (1992) 72(3) BUL Rev 463.

4
*G* [2003] UKHL 50.

5 Discussed in section 2 of this article. See also Paul Robinson and Jane Grall, ‘Element Analysis in Defining Liability: The Model Penal Code and Beyond’ (1983) 35 Stan L Rev 681.

6 A notable exception is the work of Paul Robinson. See eg *Structure and Function in Criminal Law* (OUP 1997). However, as we will explore, even Robinson’s willingness to look beyond the present-fault paradigm has (in our view) not gone far enough.

7 See Findlay Stark, ‘It’s Only Words: On Meaning and *Mens rea*’ (2013) 72 CLJ 155.

8 Hereafter ‘actions’ for brevity. We use ‘conduct’ to refer to a combination of acts, circumstances and results, and ‘actions’ to refer to physical movements and omissions only.

9 Criminal Damage Act 1971, s 1(2).

10 Criminal Attempts Act 1981, s 1.

11 Criminal Law Act 1977, s 1.

12 Serious Crime Act 2007, Part 2.

13 To explain this thesis, and demonstrate its doctrinal pay-offs, we will outline how *mens rea* terms might be defined (ie for T2 target elements) where necessary. However, although we have some established views on this, and will refer to some of our other writings where relevant, the central thesis we set out in this article is not tied to or dependent upon any single definition of any particular *mens rea* term. Rather, our thesis calls for the recognition of an increased variety of *mens rea* targets (ie those at T2), and the need for *mens rea* terms to be defined and analysed distinctly in respect to them. In short, we are concerned with identifying a conceptual space for definitional and analytical debate, not foreclosing the outcomes of such debate. One may well ask why this matters. Our answer is that it is an essential prior step to having meaningful and coherent debates about what is a justifiable *mens rea* for particular offences, which terms to use, and how to define them—ie the first inquiry noted above with which so much academic writing and judicial decision making is concerned.

14 On the ‘correspondence principle’, see Jeremy Horder, ‘A Critique of the Correspondence Principle’ [1995] Crim LR 759; Barry Mitchell, ‘In Defence of a Principle of Correspondence’ [1999] Crim LR 195.

15 For wider discussion, see John Gardner and Heike Jung, ‘Making Sense of *Mens rea*: Antony Duff’s Account’ (1991) 11 OJLS 560.

16 See eg definitions in the US MPC 1962, § 2.02(2) and the Law Commission’s Draft Criminal Code for England and Wales Bill 1989 (E&W DCC), s 8.

17 See eg definitions within the US MPC and E&W DCC. This is also true of all major textbooks, which, at best, include only subsections considering how *mens rea* is understood in the context of ulterior fault. One particularly telling example is the excellent book on the meaning of intention by Antony Duff (*Intention, Agency and Criminal Liability* (OUP 1990)), which contains only the barest references to intention outside of the ‘in action’ present-fault paradigm, at 17 and 56.

18
*Saik* [2006] UKHL 18.

19 ibid [20].

20 ibid [25] and [119].

21 ibid [101]–[102].

22 ibid [59].

23 (1978) 67 Cr App R 131, discussed further in section 3 of this article.

24
*Jogee* [2016] UKSC 8, discussed further in sections 3 and 4 of this article.

25 Criminal Attempts Act 1981, s 1. We are assuming an incomplete attempt here, where D knows that further conduct is required to complete the principal offence attempted.

26 Criminal Law Act 1977, s 1. We are assuming a conspiracy where D agrees to complete the future offence herself, rather than (or in combination with) D2.

27 eg possession of anything with intent to damage property (Criminal Damage Act 1971, s 3); possession of a controlled drug with intent to supply it to another (Misuse of Drugs Act 1971, s 5(3)); possession of a firearm or ammunition with various intent as to future offending or endangerment (Firearms Act 1968); even theft, where an intention to permanently deprive often involves future-focused intent to retain possession or destroy (Theft Act 1968, s1 ).

28 Beyond s 9(1)(a) burglary, this includes trespass with intent to commit a sexual offence (Sexual Offences Act 2003, s 63), unauthorised access (to a computer) with intent to commit further offences (Computer Misuse Act 1990, s 2), etc.

29 Including general preparation offences, such as s 5 of the Terrorism Act 2006, engaging in any conduct in preparation for giving effect to an intention to commit an act of terrorism.

30 Allowing for pre-emptive criminalisation where D’s T1 conduct may otherwise appear objectively innocent, as well as cases where, although D commits a lesser crime, the full extent of her intended wrong warrants a more serious label and punishment. See Jeremy Horder, ‘Crimes of Ulterior Intent’ in Andrew Simester and Tony Smith (eds), *Harm and Culpability* (OUP 1996) 153. Horder argues for their increased and more systematic use across the criminal law.

31 Certain T2 offences, such as theft, will also require ulterior *mens rea* at T2. We do not specifically address such elements here (though we have done so in detail elsewhere, see JJ Child, ‘The Structure, Coherence and Limits of Inchoate Liability: The New Ulterior Element’ (2014) 34 LS 537), but simply note that their analysis should follow the same approach that we provide for T2 circumstances and results.

32 See definitions of voluntary action in the US MPC § 2.01. Many commentators present voluntariness as part of the *actus reus*. The choice of categorisation does not matter for present purposes.

33 Robinson recognises this within his excellent work on the structure of crimes. However, focusing on functional roles, he does not go on to explore the full extent of the definitional problems that arise from it, as we do below. Robinson (n 6) ch 6.

34 Note that although current definitions of voluntariness do not fit the T2 action target, it would be possible to redefine ‘voluntariness as to T2 actions’ distinctly from ‘voluntariness as to T1 actions’. However, as the common law already marks the distinction with the use of the term ‘intention’ for the former, we will adopt the same language.

35 Discussed in section 2 of this article.

36 See JJ Child, ‘Understanding Ulterior *Mens rea*: Future Conduct Intention is Conditional Intention’ (2017) 76(2) CLJ 311.

37 eg where D decides to kill V if he fails to pay a debt, we have a ‘conditional intention’ for future action. However, when later killing V, D’s intention in-action is necessarily non-conditional: D’s action demonstrates that any previous conditions on that action have been resolved or removed.

38 Child (n 36).

39 For a summary and useful analysis of these cases, see Kenneth Campbell, ‘Conditional Intention’ (1982) 2 LS 77, 78–82; Laurence Koffman, ‘Conditional Intention to Steal’ [1980] Crim LR 463.

40 It was this mismatch that led to the rather awkward expression quoted earlier, from Lord Scarman in *Husseyn* (n 23), that ‘it cannot be said that one who has it in mind to steal only if what he finds is worth stealing has a present intention to steal’.

41 Discussed in Glanville Williams, ‘The Three Rogues Charter’ [1980] Crim LR 263.

42
*AG’s Reference (Nos 1 and 2 of 1979)* [1980] QB 180.

43 Moving beyond the early attempt and burglary cases, we see particular discussion of conditional intention in conspiracy cases. See eg *O’Hadhmaill* [1996] Crim LR 509.

44 US MPC, § 2.02(6).

45 Conditional intention could be reinterpreted as equivalent to the German *dolus eventualis*, but this would require a fundamental reconception of T1 *mens rea* (and is arguably unnecessary alongside criminal recklessness).

46 [2016] UKSC 8, [2016] UKPC 7.

47 ibid [92].

48 See eg Andrew Simester, ‘Accessory Liability and Common Unlawful Purposes’ (2017) 133 LQR 73; *Miller* [2016] HCA 30; *Chan Kam Shing* [2016] HKCFA 87.

49 See JJ Child and GR Sullivan, ‘The Current State of Murder in English Law’ in Alan Reed and others (eds), *Homicide in Criminal Law* (Routledge 2018) 62, 75–7. Beyond complicity, the conditional intention problem demonstrates several important points within the wider context of this article. This includes the basic problem of using present-fault definitions of *mens rea* terms when targeting elements at T2 that we examine throughout. But also, beyond this, it cautions against the assumption that this problem can be solved by adapting the present-fault definition (ie with general glosses to the meaning of *mens rea* terms) rather than accepting that distinct targets may require distinct *mens rea* definitions.

50 We briefly discuss cases of impaired capacity as a result of intoxication in the concluding remarks.

51 As Bratman memorably states, ‘my intention today does not reach its ghostly hand over time and control my actions tomorrow’: Michael Bratman, *Intention, Plans, and Practical Reason* (CSLI 1999) 5.

52 Developed in Child (n 36). On commitment to future action, see also Bratman (n 51).

53 This ranges from awareness of conditions, usefully discussed in John Cartwright, ‘Conditional Intention’ (1990) 60 Philosophical Studies 233, to more complex accounts of competing intentions and practical reasoning, such as Gideon Yaffe, ‘Conditional Intent and *Mens rea*’ (2004) 10 Legal Theory 273.

54 See the discussion in Gregory Klass, ‘Conditional Intent to Perform’ (2009) 15 Legal Theory 107; Larry Alexander and Kimberly Kessler, ‘*Mens rea* and Inchoate Crimes’ (1997) 87 J Crim L & Criminology 1144; Larry Alexander and Kimberly Kessler-Ferzan, *Crime and Culpability* (CUP 2009) 204–5. See also *Reed* [1982] Crim LR 819, where the court suggests ‘peripheral’ conditional intentions may not be legal intentions.

55 The concern here is that a subjective condition could mean the deferral of a decision to act at T2 rather than a conditional commitment to doing so. See J Parry, ‘Conditional Intention (1) A Dissent’ (1981) Crim LR 6; see also the brief discussion in Glanville Williams, *Textbook of Criminal Law* (Stevens 1978) 652.

56 Each is discussed in Child (n 36).

57 The Law Commission has come remarkably close to the same conclusion, though without recognising the associated definitional error within current approaches to intention: ‘“intention” to do something need not involve a high-level commitment, unlike a pledge, vow or oath. In this context, an “intention” is nothing more than a (possibly, quite weak) provisional conclusion reached in reasoning about action’: Law Commission, *Conspiracy and Attempts* (Law Com No 318 (n 75), 2009) 2.113.

58 Their solutions, though not all the same in substance, are of a particular type. They involve identifying apparent *mens rea* definitional problems presented by using present-fault terms as to future T2 targets and modifying or avoiding such definitions in response.

59 See eg Stephen Shute, ‘Knowledge and Belief in the Criminal Law’ and GR Sullivan, ‘Knowledge, Belief, and Culpability’ both in Stephen Shute and Andrew Simester (eds), *Criminal Law Theory* (OUP 2001). They contend that the truth requirement can be inferred or assumed in *certain* cases to allow for knowledge as to future circumstances, a position accepted (for practical purposes at least) in leading cases such as *Saik* (n 18). cf criticism of this position in David Ormerod, ‘Making Sense of *Mens rea* in Statutory Conspiracies’ (2006) 59 CLP 185, 208–9, with support from cases such as *Sunair Holidays* [1973] 1 WLR 1105 and *Harmer* [2005] EWCA Crim 1. The confusion here is neatly encapsulated in the E&W DCC, which, having defined ‘knowledge’ in terms of true belief and acknowledging in the commentary that in the ‘strictest sense’ future facts cannot be true at T1, nevertheless states that knowledge can be applied to future circumstances (cl 18(a)).

60 Including that a risk is unreasonable to run for ‘recklessness’, that behaviour is ‘dishonest’ in the circumstances it is conducted, and so on.

61 In this section, we are concerned with conspiracies where D agrees to complete the future principal offence herself. We discuss cases where D agrees to D2 completing the principal offence in section 4 of this article.

62 Criminal Law Act 1977, s 1. s 1(2) clarifies that ‘a person shall nevertheless not be guilty of conspiracy to commit that offence by virtue of subsection (1) above unless he and at least one other party to the agreement intend or know that that fact or circumstance shall or will exist at the time when the conduct constituting the offence is to take place’.

63 See *Rivzi* [2003] EWCA Crim 3575; *Sakavickas* [2004] EWCA Crim 2686; *Harmer* [2005] EWCA Crim 1.

64 Above n 18.

65 Ormerod (n 59).

66 ibid 206.

67 This is expressed using examples of conspiracy to launder foreseeing that property might be stolen, as well as conspiracy to rape foreseeing that V may not consent. ibid 224.

68
*Saik* (n 18) [26].

69 The Law Commission provide useful analysis of the inconsistent approaches to these terms in *Conspiracy and Attempts* (Law Com CP No 183, 2007) 4.70–4.93. cf Stark (n 7). Stark commends the court for not moving too far from definitions of knowledge within the present-fault paradigm (174–5), correctly highlighting that to do otherwise does ‘violence to the notion of knowledge applied more generally in the criminal law’. The problem, however, is that if present-fault definitions cannot be logically applied to future conduct, their application does violence to the law of conspiracy.

70 Emphasis added.

71
*Saik* (n 18) [119].

72 ibid [25]–[26].

73 ibid [20] and [23]. Though D may believe that property is stolen in cases of this kind, it is much less clear that she would intend or want it. It is therefore questionable whether intention to act is being conflated here with intention as to the circumstance.

74 Law Com CP No 183 (n 69).

75 Law Commission, *Conspiracy and Attempts* (Law Com No 318, 2009).

76 Law Com CP No 183 (n 69) [1.22].

77 Law Com No 318 (n 75) Part 8.

78
*A v DPP* [2003] All ER 393.

79 Not a problem of intentionality.

80 Intending a T2 result at T1 creates definitional challenges, but committing to act with intention at T2 as to a T2 result allows present definitions of intention to be applied without difficulty.

81 Law Com CP No 183 (n 69) [1.22].

82 It should be remembered, however, that recklessness requires an objective element, and that it is unreasonable for D to run the relevant risk. This remains definitionally problematic, like knowledge, when applied from T1 to events at T2.

83 Foreseeing that a future action *may* result in a crime is a significantly different wrong than acting with intention/knowledge that it should/will.

84 The distinction is problematic in both descriptive terms (ie it is not always clear how such elements are distinguished) and normative terms (ie it is not clear why such elements should be distinguished). Discussed in JJ Child and Adrian Hunt, ‘*Mens rea* and the General Inchoate Offences: Another New Culpability Framework’ (2012) 63 NILQ 247.

85 See also Glanville Williams, ‘Intents in the Alternative’ (1991) 50(1) CLJ 120, where Williams struggles to explain why a similar approach should not apply to foreseen results.

86 Discussed in Law Com No 318 (n 75) Appendix B.

87 We see this, for example, in Ormerod’s formulation (n 59) 244. The problem here is that conspiracy does not *require* the events at T2 to ever arise (including circumstances), despite the fact that conspiracy is often charged in relation to completed principal offences. Any scheme that relies on established T2 facts is therefore conceptually problematic—a criticism that can also be directed at Duff’s scheme for attempts in Antony Duff, *Criminal Attempts* (OUP 1996).

88 eg consent/non-consent. Outside of sexual offending, *Jackson* [1985] Crim LR 442 provides a good example of this category of T1 uncertainty, where D’s actions (to shoot V, to illicit sympathy for V) was conditional on a decision of the court (to convict) that was not yet made.

89 eg criminal origin of property where D suspects potential police intervention.

90 eg in stealing property to make it ‘stolen’.

91 This problem was identified by Smith as early as 1977 in JC Smith, ‘Conspiracy under the Criminal Law Act 1977 (1)’ [1977] Crim LR 598, 602–5.

92 This is also the reason why a shift to ‘belief’ at T1 would not resolve the problems at issue: belief does not include objective elements, and so avoids definitional constraints, but locating belief at T1 is still, in our view, looking in the wrong place for culpability.

93 eg Hale, when discussing the conspiracy to rape hypothetical, states that there should be liability where D commits to penetration ‘*even if at the time when they go ahead they know that she is not consenting*’: *Saik* (n 18) [99] (original emphasis).

94 By the majority in *Saik* and the Law Commission.

95 eg Hale states that liability for conspiracy to launder should be found ‘provided that [D] intended to put the agreement into effect even if the property was in fact the proceeds of crime’: *Saik* (n 18) [102].

96 Law Com CP No 183 (n 69) [5.19]. Emphasis in the original.

97 In view of various procedural and evidential benefits, such as avoiding problems of duplicity. See discussion in *Saik* (n 18) [39], [41] and [123].

98 An often discussed hypothetical, taken from *Reed* (n 54).

99 We discussed this possibility in section 3A of this article.

100 Serious Crime Act 2007 (SCA), Part 2.

101 Above n 24.

102 (1986) AC 27.

103 Law Com CP No 183 (n 69).

104 Law Com No 318 (n 75).

105 Referred to as an ‘impressive disarray’ in Law Commission, *Participating in Crime* (Law Com No 305, 2007) [1.16].

106 ie P’s offence at T2, which D has assisted or encouraged. *Johnson v Youden* [1950] 1 KB 544, affirmed in *Jogee* (n 24) [9].

107 David Ormerod and Karl Laird, ‘Jogee—Not the End of a Legal Saga but the Start of a New One?’ [2016] Crim LR 543, 544.

108 Including, at one time, Law Com No 305 (n 105) Part 3.

109 Simester (n 48) 82–4.

110 Including, at one time, the Law Commission, *Assisting and Encouraging Crime* (Law Com CP No 131, 1993) [2.50].

111 Most recently *Jogee* (n 24), which despite setting out to correct a ‘wrong turn’ in the interpretation of *mens rea* as to P’s offence, provides staggeringly little detail on how such *mens rea should* be understood. We discuss problems with this case further below.

112 Conspicuous for their lack of consistency between the interpretation of such *mens rea* terms.

113 eg ‘belief’ for s 45 SCA, as well as within certain interpretations of complicity.

114 ‘Belief’ (ibid) is much less problematic because it does not include necessary objective and/or causal requirements.

115 Criminal Law Act 1977, s 1, notwithstanding *Anderson* (n 102).

116 Though, curiously, this is not made explicit within the statute. See JJ Child, ‘Exploring the *Mens rea* Requirements of the Serious Crime Act 2007 Assisting and Encouraging Offences’ (2012) 76 JCL 220.

117 Following *Jogee* (n 24). See *Crilly* [2018] EWCA Crim 168; *Brown* [2017] EWCA Crim 1870; *Johnson* [2016] EWCA Crim 1613. This interpretation was also assumed in the Australian High Court (*Miller* [2016] HCA 30) and Hong Kong Final Court of Appeal (*Chan Kam Shing* [2016] HKCFA 87).

118 Regarding conspiracy, see Law Com CP No 183 (n 69) [4.34]–[4.41]; Law Com No 318 (n 75) [2.46]–[2.56]; complicity, see Law Com No 305 (n 105) [1.49] and [3.84]–[3.91].

119 Robinson (n 6) 132. See also Paul Robinson, ‘Should the Criminal Law Abandon the *Actus Reus*–*Mens rea* Distinction?’ in Stephen Shute and others (eds), *Action and Value in Criminal Law* (OUP 1993) 187, 205.

120 See *Kennedy (No 2)* [2007] UKHL 38, quoting Williams, ‘Principals' cause, accomplices encourage…’; Glanville Williams, ‘Complicity, Purpose and the Draft Code (I)’ [1990] Crim LR 4.

121 See Simester (n 48) 82–4; Andrew Simester and others, *Simester and Sullivan’s Criminal Law* (7th edn, Hart Publishing 2019) 243–5. Even the Law Commission at one time concluded that ‘if it were sought to introduce notions of intention or purpose into a general definition of complicity liability, it would seem that the object addressed will have to be the attitude of the principal offender, rather than his actual commission of the principal offence’: Law Com CP No 131 (n 110) [2.50].

122 The problem with applying ‘standard’ definitions of intention is usefully discussed in Ormerod and Laird (n 107).

123 Robinson (n 6) 132 (emphasis added).

124 Simester and others (n 121) 339. More precisely, we are told that D’s intention as to P’s offence is ‘constituted by the knowledge that his co-conspirators intend’. We interpret this as including similar hope/knowledge as to the physical elements of P’s offence, notwithstanding their discussion of *Anderson* (n 102) 338–40, because knowledge of another’s intention *simpliciter* would lead to an unacceptably wide offence. For example, where D (undercover police officer) agrees with P that P will commit an offence but D intends to intervene before the offence is committed, it would be absurd to hold D liable for a conspiracy (despite her T1 knowledge of P’s intentions). See *Yip Chiu-Cheung* [1995] 1 AC 111.

125 Discussed in section 3B of this article.

126 It is questionable whether we can or should ever present the future decision of another as virtually certain, particularly where that decision is to commit an offence. It was partly this concern that led the Commission to reject the language of intention and knowledge in Law Com CP No 131 (n 110), stating [2.57] that ‘as the matters referred to lie in the future, the accessory’s state of mind can only at best be one of belief, foresight or suspicion.’

127 See *NCB v Gamble* [1959] 1 QB 11; discussed in Antony Duff, ‘“Can I Help You?” Accessorial Liability and the Intention to Assist’ (1990) 10 LS 165; Heidi Hurd and Michael Moore, ‘Untying the Gordian Knot of *Mens rea* Requirements for Accomplices’ (2016) 32 Social Philosophy & Policy 161.

128 Discussed in section 3A of this article.

129 ie the need to distinguish what it means for each party to intend the future offence.

130 [2016] UKSC 8, [92]: ‘it will … often be necessary to draw the jury’s attention to the fact that the intention to assist, and indeed the intention that the crime should be completed, may be conditional’.

131 Explored more fully in Simester (n 48) 84–6; Child and Sullivan (n 49) 75–7.

132 These points are explored further below.

133 The definition outlined requires considerably more analysis and defence than can and will be provided here.

134 The intention to cause such effects to P’s acts is important here, beyond an intention to perform acts of assistance or encouragement simpliciter (ie focused on D’s own conduct). To the extent that this distinction is lost or confused in complicity cases provides further illustration of the inadequate state of the current law. We see this, for example, throughout the judgments in *Jogee*. Despite its *many* drafting faults, such confusion is avoided in the SCA offences, where intention as to P’s offence is distinct from an intention to perform acts ‘capable’ of assistance or encouragement; there is also no problem of this kind in the context of conspiracy.

135 Causal language is common in the discussion of complicity and similar multi-party offences, but few present the causal relationship here as akin to results. See *Mendez* [2010] EWCA Crim 516, [18; 23]; *Luffman* [2008] EWCA Crim 1739 [40]; *Bryce* [2004] EWCA Crim 1231, [72]–[74]; see also useful discussion in Sanford Kadish, ‘Complicity, Cause and Blame’ (1985) 73 CLR 323. cf Hurd and Moore (n 127) 178: ‘accomplices are just like other causers of criminal harms’.

136 We see this, for example, in the extended definition of intention as to results provided by Robinson (n 6) 132 and discussed above. There is also useful discussion of this concern in Ormerod and Laird (n 107).

137 eg, in addition to our formulation, we might limit intention to cases where D demonstrates some further affirmation towards the outcome, such as ‘endorsement’ or ‘consent’. See Beatrice Krebs, ‘*Mens rea* in Joint Enterprise: A Role for Endorsement?’ (2015) 74(3) CLJ 480; Kadish (n 135) 406.

138 Criminal Law Act 1977, s 1.

139 See discussion in section 3B of this article.

140 Irrelevant because of definitional mismatches, such as ‘knowing’ the future, as well as normative relevance, in that D’s commitment to knowing action at T2 is of greater moral salience. Discussed in section 3B of this article.

141 Just as D may conditionally commit to future actions with various expectations (eg intending to have sex with V even if she does not consent), varying expectations can also be accommodated where D intends the future conduct of P. The latter is not a conditional intention, however, as D has no T2 agency to pre-empt and condition. Rather, it is a complete intention that encompasses multiple alternative action events, in which we ask, simply, whether one of those intended potential events amounts to the relevant principal offence.

142 Accessories and Abettors Act 1861, s 8; *Jogee* (n 24). Curiously, and unlike conspiracy, examples of the analytical error in discussion of complicity are by no means universal. Perhaps because complicity forces us in all cases to acknowledge different parties at T1 and T2, the separation of action events becomes more salient in analysis. However, there remains a need for clarification and consistency.

143 Discussed in section 3A of this article. See Ormerod and Laird (n 107); Law Com CP No 131 (n 110) [2.57]–[2.58].

144 Discussed in section 3A of this article. See Child and Sullivan (n 49) 75–7.

145
*DPP v Maxwell* [1978] 1 WLR 1350.

146 This approach is also the only way to find liability where D encourages P to have sex with V ‘even if’ she does not consent. We can describe D’s intention as a single intended action with multiple potential circumstances, or as multiple intended actions with different descriptions. Either way, D intends an event in which P knowingly commits rape.

147 Law Com No 305 (n 105) [B.101] and [3.117]–[3.122].

148 eg, in the case of conspiracy to rape, whilst it is clearly challenging to conceive of knowledge at T1 as to V’s future non-consent at T2, it is perfectly sensible to ask whether D intends to have sex with V even if he comes to know before T2 that V is not consenting.

149 See *Kennedy* (n 120). Discussed in Catarina Sjölin, ‘A Step Away from Liability—Withdrawal and Fundamental Difference’ in Beatrice Krebs (ed) *Accessorial Liability after* Jogee (Hart Publishing 2020) ch 4, VII.

150 John Gardner, ‘Complicity and Causality’ (2007) 1 Criminal Law and Philosophy 127.

151 Andrew Simester, ‘Intoxication Is Never a Defence’ [2009] Crim LR 3.

152 See Hans Crombag, JJ Child and Rudi Fortson, ‘Understanding the “Fault” in Prior-Fault Intoxication’ in Alan Reed and others, *Fault* (Routledge forthcoming).

## Notes

We would like to thank those who provided comments on an earlier draft of this article—Prof Andrew Simester, Prof Jeremy Horder, Prof Antony Duff, Prof Alex Sarch and Dr Beatrice Krebs—as well as discussion groups at Kings College London, the University of Oxford and the Hebrew University of Jerusalem.

